# Refractory Keloids and Hypertrophic Scars: Immune Dysregulation and Neuroimmune Mechanisms Underlying Treatment Failure

**DOI:** 10.3390/cells15090782

**Published:** 2026-04-26

**Authors:** Daniela Grinis, Marina Thomas, Caroline Aprigliano, Anish R. Maskey

**Affiliations:** 1 Department of Basic Science, Touro College of Osteopathic Medicine, Great Falls, MT 59405, USA; dgrinis@student.touro.edu (D.G.); mthomas33@student.touro.edu (M.T.); caprigli@student.touro.edu (C.A.); 2 Department of Pathology, Microbiology & Immunology, New York Medical College, Valhalla, NY 10595, USA

**Keywords:** keloids, hypertrophic scar, wound healing, remodeling, fibrosis, neuroimmune signaling

## Abstract

**Highlights:**

**What are the main findings?**

- Keloids and hypertrophic scars are increasingly recognized as immune-driven fibroproliferative disorders rather than isolated abnormalities in wound healing.
- Dysregulation of immune signaling pathways involving cytokines like IL-6, TNF-α, TGF-β and IL-17 contributes to prolonged inflammation and fibroblast activation.

**What are the implication of the main findings?**

- Targeting immune–fibrotic signaling feedback loops represents a promising therapeutic approach beyond conventional scar-directed treatments.
- Emerging therapies aimed at modulating immune pathways can improve long-term outcomes and reduce recurrence rates.

**Abstract:**

Keloids and hypertrophic scars are fibroproliferative disorders of wound healing characterized by excessive extracellular matrix deposition, constant inflammation, and high recurrence rates despite appropriate management. Conventional therapies, including surgical excision, corticosteroid injections, laser therapy, and radiation, can provide temporary relief. However, treatment failure remains common, specifically in refractory keloids. Recent findings suggest these outcomes cannot be fully explained by technical or mechanical factors alone, and pathological scarring may reflect underlying immune and neuroimmune dysfunction. Current evidence shows prolonged activation of pro-inflammatory and pro-fibrotic cytokine pathways like IL-6, TNF-α, TGF-β, and IL-17 drives sustain fibroblast activation and disrupts normal wound healing and remodeling. Additionally, the skin functions as an integrated neuro-endocrine-immune organ, allowing bidirectional communication between cutaneous nerves, immune cells, and stromal tissue. Neurogenic inflammation is mediated by neuropeptides, mast cell activation, and stress-induced hypothalamic–pituitary–adrenal axis dysregulation, which further amplifies inflammation within scar tissue. Psychiatric comorbidities like depression, anxiety, and chronic psychological stress serve as a positive feedback mechanism and are increasingly recognized as biologically active contributors to immune dysregulation. This review highlights critical gaps in current management strategies and emphasizes the need for biologically informed, multidisciplinary approaches to improve long-term outcomes for keloid and hypertrophic scar management.

## 1. Introduction

Keloids and hypertrophic scars are fibroproliferative disorders of wound healing characterized by excessive collagen and extracellular matrix deposition and persistent inflammation after a cutaneous injury [1]. Generally, hypertrophic scars stay within the boundaries of the original wound, as compared to keloids, which extend beyond the margins of the initial injury and often demonstrate progressive growth without spontaneous resolution [2]. Patients frequently deal with symptoms like pain, pruritus, functional limitation, and cosmetic deformity, resulting in a significant reduction in quality of life compared to other chronic inflammatory skin conditions [3]. The disease burden is significantly higher in individuals with darker skin characteristics and in high-tension anatomical regions like the chest, shoulders, and earlobes, suggesting there are contributions from both biological and mechanical forces [1].

Many ways exist to manage keloids and hypertrophic scars, including surgical excision, intralesional corticosteroid injections, laser therapy, radiation, silicone gel sheeting, pressure therapy, and cryotherapy [4]. Despite the widespread use of these interventions, recurrence rates remain high, specifically for keloids, with surgical excision alone consistently associated with substantial risk of recurrence and even multimodal approaches demonstrating limited long-term durability [5,6]. Most importantly, treatment failure usually occurs despite technically appropriate procedures and adherence to established clinical protocols, suggesting that refractory disease cannot be fully explained by procedural inadequacy alone [7].

Traditional research on scarring has emphasized the process of localized excessive fibroblast deposition, disproportionate collagen synthesis, and mechanical tension as the primary drivers of disease [8]. As a result, therapeutic strategies have mainly concentrated on physical scar removal or mechanical collagen production suppression [1]. While these approaches can temporarily reduce scar volume or symptoms, they often fail to prevent recurrence, indicating the presence of deeper physiological and immunological processes responsible for the fibrotic response [9]. Emerging evidence from mechanistic and clinical studies indicates that refractory keloids and hypertrophic scars may be considered manifestations of immune dysregulation, characterized by prolonged, increased proinflammatory cytokine signaling and constant immune cell activation within scar tissue [10]. Increased levels of pro-inflammatory and pro-fibrotic cytokines like IL-6, TNF-α, and TGF-β promote fibroblast activity and excessive extracellular matrix formation and deposition, adding to the chronicity and treatment resistance [11]. Beyond the local immune dysregulation, the skin serves as a dual active neuroimmune organ that can maintain a bidirectional communication with the nervous system [12]. It is important to note that both skin and the nervous system are derived from a common embryonic progenitor, the ectoderm, providing a biological basis for a lifeline neuroimmune–cutaneous interaction. Psychological stress and neuroendocrine signaling can further amplify immune activation through upregulation of stress-response pathways, reinforcing fibrotic signaling and impairing wound healing [13]. Therefore, recognizing refractory keloids and hypertrophic scars as manifestations of immune and neuroimmune dysregulation, rather than mere dermatological anomalies, underscores the limitations of conventional therapies and elucidates the need for targeted, mechanism-based treatment strategies. This narrative aims to synthesize evidence on the immunologic and neuroimmune mechanisms underlying keloid and hypertrophic scar formation, with a focus on immune–fibrotic interactions, treatment resistance, and emerging therapeutic targets [14].

## 2. Methods

A literature review was conducted to evaluate the immunologic, neuroimmune, and psychosocial mechanisms contributing to refractory keloids and hypertrophic scars. Databases, including PubMed and Google Scholar, were systematically searched for relevant studies published within the past 20 years. Search terms include “keloids”, “hypertrophic scars”, “immune dysregulation”, “neuroimmune signaling”, “wound healing”, “psychological stress”, and “fibrotic disorders”. Priority was given to peer-reviewed clinical studies, mechanistic research, and review articles addressing inflammatory signaling pathways, neuroimmune interactions, and therapeutic outcomes. Articles were selected based on relevance to the scope of this review and their contribution to understanding of the underlying biological mechanisms and clinical implications. No formal systematic review protocol was applied, as the aim of this narrative review is to provide a focused, mechanistic and translational overview of the topic.

## 3. Overview and Clinical Challenges

Keloids and hypertrophic scars are both forms of pathological scarring arising from abnormal wound-healing processes; however, when comparing both, they are different in growth behavior, chronicity, and clinical course [14]. Although keloids and hypertrophic scars share overlapping features of abnormal wound healing and fibrosis, they differ significantly in growth behavior, recurrence patterns and behavior, and potentially underlying immune pathophysiology. Keloids are characterized by continued growth beyond the normal wound borders with higher recurrence rates, while hypertrophic scars usually remain confined to the original injury and may regress over time. Hypertrophic scars are characterized by excessive collagen deposition that remains within the original wound margins and either stabilizes or regresses over time with the remodeling phase of the healing process. On the other hand, keloids tend to extend beyond the original margins and are characterized by progressive growth without resolution [7]. Therefore, keloids are known to be the more aggressive and chronic form of fibroproliferative disorder, often associated with recurrent inflammation and treatment resistance [15]. According to epidemiologic data, keloids disproportionately affect individuals with darker skin phenotypes, with the highest prevalence reported among African, Asian, and Hispanic populations [16]. Additionally, areas prone to increased skin tension, such as the anterior chest, shoulders, upper back, and earlobes, have excessive collagen deposition and are associated with increased risk of keloid and hypertrophic scar development [17]. In addition, genetic susceptibility, age, anatomic location, and wound-related factors all contribute to disease risk, highlighting the multifactorial nature of pathological scarring [18]. Population-based studies suggest a genetic predisposition, specifically among individuals of African, Asian, and Hispanic descent. For instance, polymorphisms affecting genes involved in collagen synthesis, transforming growth factor-β (TGF-β) signaling, and inflammatory cytokine regulation have been linked to abnormal fibroblast activation and excessive extracellular matrix deposition during the tissue healing process. These genetic variations can potentially influence wound healing signaling balance, fibroproliferative responses, and recurrence risk, further emphasizing the multifactorial aspect of pathological scarring [18,19].

A variety of therapeutic options are currently available on the market and being used in the management of keloids and hypertrophic scars, including surgical excision, intralesional corticosteroid injections, laser-based therapies, radiation therapy, silicone gel sheeting, pressure therapy, and cryotherapy [4] (Table 1). All these approaches primarily target reducing scar volume, either by reducing scar volume, suppressing fibroblast activity locally in the tissue, or mechanically altering the wound micro-environment [20]. While these therapeutic modalities have the potential to achieve partial or temporary improvement in scar appearance or symptoms, outcomes are highly variable between patients, and successful treatment is often not achieved with many conventional approaches [9]. Recurrence of keloids and hypertrophic scars despite different therapeutic modalities remains a main challenge for clinicians. Surgical excision alone carries a high risk of regrowth, and even when combined with additional therapies, recurrence rates remain substantial, with considerable variability in patient-to-patient response [5].

This heterogeneity highlights complex biological differences in inflammatory and fibrotic signaling between individuals [10]. Altogether, these limitations highlight the need to move beyond purely procedural techniques and toward a mechanistic understanding of the immune and neuroimmune processes driving refractory disease pathophysiology.

## 4. Immune Dysregulation in Pathological Scar Formation

**Figure 1:**
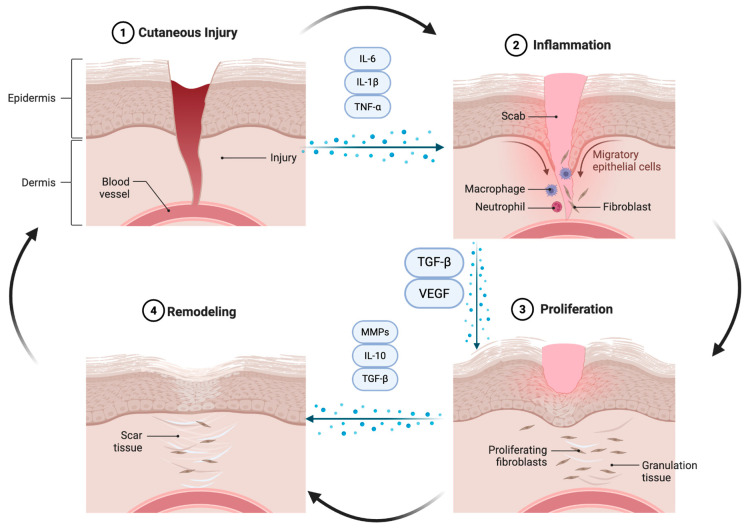
Phases of normal cutaneous wound healing. Following cutaneous injury, wound repair progresses through coordinated and controlled phases. (1) Hemostasis and early injury initiate clot formation and release of growth factors that recruit immune and promote healing. (2) During inflammation, neutrophils and macrophages infiltrate the wound and secrete pro-inflammatory cytokines (e.g., IL-1β, IL-6, TNF-α) to clean debris and pathogens. (3) The proliferative phase is characterized by fibroblast proliferation, angiogenesis, and granulation tissue formation, with extracellular matrix deposition supported by growth factors such as VEGF and TGF-β. (4) During remodeling, matrix metalloproteinases (MMPs) and anti-inflammatory mediators (e.g., IL-10) regulate collagen production and extracellular matrix turnover, resulting in restoration of normal tissue architecture. Arrows indicate progression through the wound-healing cascade. Created in BioRender. Created by (Grinis, D.) (2026) https://BioRender.com/jhyay34.

Normal cutaneous wound healing proceeds through tightly regulated immune-mediated, proliferative, and remodeling phases (Figure 1). In keloid and hypertrophic scar formation, this tightly regulated signaling process is disrupted by a prolonged and exaggerated inflammatory phase, characterized by excessive immune activation and persistent fibroblast stimulation rather than timely resolution [1]. Molecular studies consistently demonstrate increased immune cell infiltration, elevated pro-inflammatory cytokine expression, and failure to downregulate inflammatory signaling within pathologic scar tissue microenvironment [10]. A defining feature of pathological scarring is the failure to transition from the proliferative phase to normal extracellular matrix remodeling. Instead of undergoing collagen remodeling and scar maturation, fibroblasts within keloids and hypertrophic scars remain constantly active, producing excessive and disorganized collagen [7]. Prolonged fibroblast activation in keloids and hypertrophic scars is sustained through dysregulated pro-fibrotic signaling pathways, involving transforming growth factor-β (TGF-β) and interleukin-6 (IL-6). TGF-β signaling, primarily through the SMAD2/3 pathway, stimulates fibroblasts to myofibroblast differentiation, enhances extracellular matrix production, and inhibits apoptosis, perpetuating sustained fibroblast activity. Additionally, IL-6–mediated activation of the JAK/STAT3 pathway further drives fibroblast survival. These pathways form a positive feedback loop in which activated fibroblasts constantly produce cytokines and growth factors, maintaining a self-activation state. This self-amplifying cycle prevents normal transition into the remodeling phase of wound healing, contributing to chronic fibrosis and treatment resistance [21]. This abnormal wound-healing process reflects not only a local fibroblast abnormality but an ongoing inflammatory microenvironment that continuously reinforces fibrotic and inflammatory signaling [9].

Interleukin-6 (IL-6) plays a central role in connecting the inflammatory and fibrotic pathways in the pathological scarring process. Elevated IL-6 expression has been consistently identified in keloid tissue and primary human keloid-derived fibroblasts compared with normal skin and non-pathological scars [9]. IL-6 has a variety of downstream effects and functions, including fibroblast proliferation, resistance to apoptosis, and increased collagen synthesis, therefore maintaining fibrotic activity beyond the normal wound-healing timeline [9]. Additionally, the IL-6 signaling pathway also amplifies downstream immune activation, resulting in a chronic inflammatory state that favors scar persistence [15]. Similarly, tumor necrosis factor-α (TNF-α) is another key mediator in keloid pathogenesis. While TNF-α plays a pivotal role in early wound formation, its persistent expression adds to the chronic inflammatory process and immune dysregulation within scar tissue [22]. High levels of TNF-α have been associated with keloid lesions and are known to promote immune cell recruitment, sustain inflammatory signaling, and indirectly enhance fibroblast activation [22]. This prolonged upregulated inflammatory response creates a positive feedback loop in which immune activation and fibrosis mutually drive and enhance one another. Additionally, transforming growth factor-β (TGF-β) is known to be a pro-fibrotic cytokine involved in extracellular matrix signaling and fibroblast differentiation. Specifically, in keloids and hypertrophic scars, overexpression of TGF-β signaling contributes to excessive collagen deposition and impaired matrix remodeling [23]. Nevertheless, TGF-β signaling alone does not fully explain the chronic inflammation and treatment resistance observed in keloid disease, as its fibrotic effects are closely modulated by the microenvironment [23]. TGF-β is part of a broader immune–fibrotic network response rather than a solitary driver causing disease pathogenesis. Additionally, experimental evidence supports the role of persistent immune signaling in maintaining fibroblast activation [22,24]. In vitro studies have shown that elevated IL-6 levels promote fibroblast differentiation, apoptosis resistance, and increased collagen synthesis compared to normal dermal fibroblasts [11]. Moreover, mechanistic studies have demonstrated that TGF-β–driven signaling pathways contribute to fibroblast activation and mesenchymal transition processes that reinforce fibrosis [21,25]. In vivo and translational studies further support these findings, demonstrating increased immune cell infiltration and cytokine-driven fibroblast activation within keloid lesions, reinforcing the link between chronic inflammation and fibrosis [22,24]. In addition to persistent fibroblast activation, alterations in collagen remodeling represent a key feature of pathological scarring. Histological analysis, including Masson’s trichrome staining and polarized light microscopy, allows evaluation of collagen fiber organization, density, and alignment within scar tissue. Additionally, molecular markers such as matrix metalloproteinases (MMPs) and their tissue inhibitors (TIMPs) are used to assess extracellular matrix turnover and remodeling dynamics. In keloids and hypertrophic scars, dysregulation of MMP activity contributes to impaired collagen degradation and excessive matrix accumulation [26,27].

**Figure 2:**
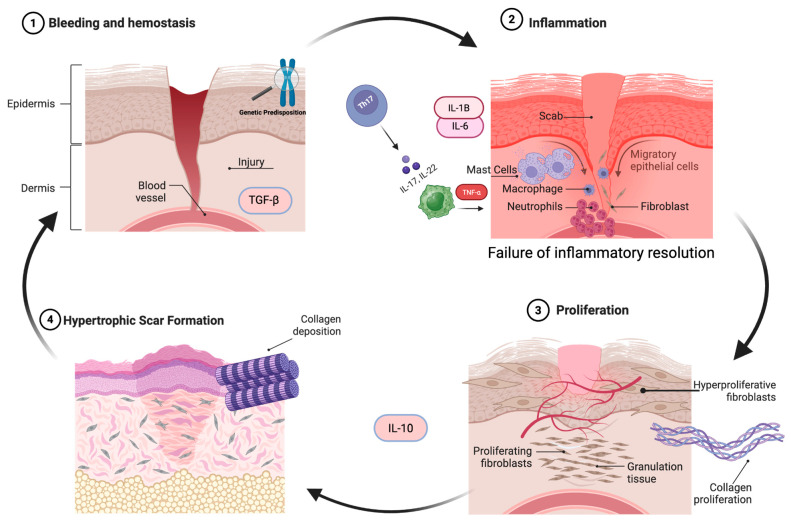
Immunologic and fibrotic mechanisms driving hypertrophic scar and keloid formation. In genetically predisposed individuals, wound healing deviates toward pathological fibrosis. (1) Injury and hemostasis initiate TGF-β release and early signaling events that activate repair pathways. (2) The inflammatory phase is marked by sustained immune activation and inflammatory signaling. (3) During proliferation, persistent cytokine and growth factor signaling (e.g., TGF-β, VEGF) drive fibroblast hyperproliferation, angiogenesis, and extracellular matrix synthesis, including collagen deposition. (4) Failure of inflammatory resolution leads to hypertrophic scar formation characterized by dense collagen deposition and prolonged fibroblast activity. Anti-inflammatory mediators like IL-10 are insufficient to restore normal tissue remodeling. Arrows indicate progression toward fibrotic scar formation. Created in BioRender. Created by (Grinis, D.) (2026) https://BioRender.com/e5n0eit.

Recent experimental research has also highlighted the role of the TH17/IL-17 axis as a potential contributor to pathological scarring formation. Studies have shown that IL-17-driven inflammation is not only associated with keloid formation but also contributes to disease progression. Studies have demonstrated that IL-17–driven inflammatory signaling promotes fibroblast activation and maintains a pro-fibrotic microenvironment, while disruption of this pathway has been associated with reduced inflammatory signaling and fibrotic responses [26,28]. IL-17 signaling and Th17 cell infiltration have been identified in keloid tissue, suggesting the possibility of Th17 playing a central role in keloid pathogenesis [28]. IL-17 further amplifies pro-inflammatory cytokines, enhances collagen production, and therefore links adaptive immune responses to fibrotic progression. (Figure 2). These findings further support the concept that keloid formation is an immune-driven disorder and not purely a mechanical or fibroblast autonomous process.

Although keloids and hypertrophic scars present as localized cutaneous lesions, the immune signaling driving their persistence extends beyond the visible scar tissue [10]. The complex interaction between multiple pathways, including pro-inflammatory cytokines, immune cells, and fibroblast–immune interactions, forms a convoluted network that cannot be fully disrupted by localized procedural interventions alone [29]. This helps explain why treatments aimed solely at scar removal or collagen reduction frequently fail and result in recurrence despite technically being a suitable procedure. Within scar tissue, immune dysregulation provides a biological explanation for resistance to localized therapies. Therefore, we must address the underlying inflammatory mechanisms and immune–fibrotic signaling environment and acknowledge the fact that procedural approaches can transiently reduce scar burden but fail to achieve a full-term resolution [29]. Recognizing pathological scarring as an immune response process, therefore, represents a critical shift in understanding treatment failure and informs the need for biologically targeted multifactorial management therapies.

In addition to cytokine signaling, multiple cell types interact within the scar microenvironment to drive prolonged pathological fibrosis. Fibroblasts function as a pivotal component, responding to pro-inflammatory cytokines by increasing collagen production and resisting apoptosis. Immune cells, including macrophages and T cells, further enhance this inflammatory response through sustained cytokine release. Mast cells have been linked as key contributors, releasing histamine, proteases, and pro-fibrotic mediators that enhance fibroblast activation and extracellular matrix deposition. Furthermore, recent emerging evidence proposes that sensory neurons participate in this network through neuropeptide signaling, linking neural input to immune activation. Altogether, these interactions form a complex cellular network that reinforces chronic inflammation and fibrotic progression in pathological scarring [9,22,30,31,32,33].

## 5. The Skin–Brain–Immune Axis in Refractory Scarring

Skin is increasingly recognized as an integrated neuro-endocrine–immune organ rather than a passive barrier tissue. The embryonic ectoderm serves as a precursor to both the epidermis and the nervous system, providing biological plausibility for lifelong bidirectional communication between cutaneous sensory pathways and immune activity [34]. Cutaneous innervation allows neural signals to rapidly shape local inflammation and tissue remodeling through neuropeptide signaling pathways as well as neuroimmune signaling circuits [35].

The skin contains a dense network of sensory nerve fibers that connect directly with immune cells and stromal cells, creating a functional neuroimmune unit in peripheral tissues [30]. The crosstalk between the cutaneous system and the neuroimmune network is mediated by neuropeptides and inflammatory cytokines that act bidirectionally [30]. For instance, substance P and CGRP are neuropeptides released from cutaneous nerves and promote vasodilation, immune cell recruitment, and mast cell activation, amplifying local inflammatory responses in the dermis [31]. Additionally, mast cell activation is a key amplifier of neurogenic inflammation because mast cells release histamine, cytokines, proteases, and vasoactive mediators that further intensify immune signaling and tissue-level inflammation [32]. In addition to immune activation, neuropeptides can directly influence stromal remodeling, with substance P demonstrated to enhance collagen remodeling and change matrix-regulatory gene expressions through neurokinin-1 receptor signaling in human cells [33]. The neuroimmune and stromal signaling pathways provide a mechanistic basis for how neural signaling can create a positive feedback loop between inflammatory and fibrotic pathways associated with pathological scar persistence [30].

An important, but often overlooked, aspect of wound biology is psychological stress, with clinical and experimental evidence showing that stress-related neuroendocrine signaling can impair immune regulation and delay normal wound healing [36]. Stress activates the hypothalamic–pituitary–adrenal axis and sympathetic nervous system, shifting cytokine signaling and immune-cell migration in different ways that can prolong inflammation rather than resolution [36]. Moreover, stress also increases peripheral neuropeptide signaling pathways and secretion, including substance P, which serves as a neuroimmune mediator known to amplify inflammatory communication pathways via paracrine and endocrine-like mechanisms between the cutaneous and neuroimmune systems [37]. Since neurogenic inflammation can amplify the local cytokine release and immune activation, chronic or repeated stress induction have the possibility to promote a wound environment that favors prolonged inflammation and disproportionate remodeling [31]. Psychosocial stress has been associated with keloid formation and may contribute to immune dysregulation by stimulating pro-inflammatory and pro-fibrotic signaling pathways rather than acting solely through behavioral factors [38]. This framework provides a biological explanation for the clinical observations that scars can worsen or have the potential to recur during periods of stress, specifically in individuals predisposed to upregulation of immune–fibrotic pathways [36].

## 6. Psychiatric and Psychological Factors as Biological Modulators

Patients with keloids encounter disproportionately high levels of depression and anxiety as compared to patients with other chronic dermatological disorders [1,3]. Beyond reduced quality of life, depression and anxiety are being increasingly recognized as biologically active disease contributors rather than purely independent psychosocial consequences [39]. Both conditions are associated with chronic low-grade systemic inflammation with elevated pro-inflammatory cytokines like IL-6, TNF-α, and CRP, and overlap with cytokine profiles seen in scarring conditions [40]. These elevated levels of pro-inflammatory cytokines and the profile overlap between psychiatric and chronic dermatological disorders provide a possible link between psychiatric comorbidities and immune dysregulation within keloid tissue [10].

After initiation of psychological stress, the hypothalamic–pituitary–adrenal (HPA) axis and sympathetic nervous system, leading to continuous neuroendocrine signaling that disrupts immune homeostasis [36]. Chronic HPA axis activation changes cortisol signaling pathways and glucocorticoid receptor sensitivity threshold, promoting immune activation rather than resolution [41]. Stress-induced neuroendocrine dysregulation promotes pro-inflammatory cytokine signaling and production as well as immune cell recruitment, reinforcing the positive feedback loop within the inflammatory environment of scar tissue [12]. For keloids, the positive feedback of stress immune response can sustain prolonged fibroblast activation and extracellular matrix deposition beyond the normal wound-healing timeline [22]. Furthermore, psychiatric comorbidities can act as an initiating event that shifts the immune system towards an elevated inflammatory signaling, lowering the threshold for excessive fibrotic responses after cutaneous injury [15]. As a result, psychiatric and psychological factors can help explain the variability in response to treatment and recurrence rates observed between patients with otherwise similar keloid characteristics [14]. 

## 7. Discussion

Even though there are many available mechanical procedures on the market for interventions for keloids and hypertrophic scars, recurrence remains common even when techniques are applied correctly and in combination. This pattern suggests that treatment failure is not fully explained by inadequate technique but rather reflects persistent biological drivers that remain overlooked [1,7]. Pathological scarring is being increasingly recognized as a symptom of sustained and prolonged inflammation with elevated pro-inflammatory cytokines and failure of normal wound healing. Localized procedures have the potential to reduce scar volume or symptoms; however, they do not address the core issue of the immune–fibrotic feedback loops that promote fibroblast activation and extracellular matrix deposition [10]. Moreover, procedural trauma itself may act as a secondary inflammatory response in immunocompromised individuals, re-activating immune pathways and lowering the threshold for recurrence of fibrotic responses [22].

Recent evidence supports the concept that keloids represent immune-driven fibroproliferative disorders rather than isolated fibroblast abnormalities. High levels of pro-inflammatory and pro-fibrotic cytokines, including IL-6, TNF-α, TGF-β, and IL-17, have been linked to the pathogenesis of keloid tissue, promoting a positive feedback loop for chronic immune activation and prolonged fibroblast proliferation [9,28].

Psychological stress, depression, and anxiety are disproportionately prevalent among patients with keloids and hypertrophic scars and are increasingly recognized as biologically relevant factors that may contribute to disease persistence rather than only psychosocial consequences [3,40]. These conditions are associated with chronic low-grade systemic inflammation and cytokine profile shifts similar to those observed in pathological scar formation [3,40]. Activation of the hypothalamic–pituitary–adrenal (HPA) axis and sympathetic nervous system changes cortisol signaling pathways and glucocorticoid receptor sensitivity, promoting prolonged immune activation and increasing inflammatory signaling within the scar microenvironment [36]. Chronic stress-induced neuroendocrine dysregulation can therefore contribute to prolonged fibroblast proliferation and signaling beyond normal wound-healing timelines [36,38]. Despite growing recognition of these associations, current research refers to psychological stress as a consequence of scars rather than a mechanistic contributor to immune dysregulation and scar persistence [38]. Mechanistic investigations directly linking psychiatric stress to immune signaling alterations, pro-inflammatory cytokine upregulation, and fibroblast proliferation within scar tissue remain limited, and the role of neuroendocrine pathways in pathological scarring is still lacking characterization [36,38].

This gap emphasizes the current limitations in management approaches. The complex interaction between immune dysfunction, neuroendocrine signaling, and tissue reinforces the inadequacy of single-modality treatment strategies for refractory keloids [12]. Incorporating stress and psychiatric screening into clinical practice, as well as adopting multidisciplinary approaches, can help explain the patient variability in treatment response [12,39]. Recognition of the skin as a neuro-endocrine–immune organ supports an inclusive therapeutic approach and may provide a critical foundation for improving long-term outcomes in refractory scarring [12]. Genetic susceptibility can contribute to variability in disease severity, recurrence rate, and treatment response. Polymorphisms affecting collagen synthesis, TGF-β signaling, and inflammatory cytokine levels can promote excessive response of fibroblast proliferation and constant prolonged inflammation, predisposing certain individuals to refractory scarring despite the correct therapeutic approach. Recognition of genetic influences can improve risk stratification and support development of targeted therapeutic approaches [18,19].

Most of the currently available literature on keloids and hypertrophic scars relies on a narrow examination of tissue sampling, concentrating mainly on the inflammatory response [42,43]. Such designs fail to consider the dynamic immune changes that occur across wound healing, treatment interventions, and recurrence, particularly in refractory keloid disease. The absence of long-term immune profiling limits the ability to differentiate the initiating mechanism behind the prolonged inflammatory response, reflecting the complex immune interactions that drive fibrotic disease processes [21]. The inability to capture this heterogeneity also prevents identification of biomarkers predictive of disease recurrence [9,19]. Despite the growing recognition of the skin as a neuro-endocrine–immune organ, psychoneuroimmunology remains overlooked in pathological scar research. The current available models of keloid formation address the main immunological contributors while failing to take into account the neuroendocrine contribution to immune persistence [12].

Collectively, the available evidence supports an integrated model in which keloid and hypertrophic scar formation arise from constant immune activation coupled with dysregulated neuroimmune signaling, resulting in prolonged fibroblast activation and extracellular matrix deposition. Rather than representing isolated abnormalities in wound healing, these findings suggest that pathological scarring reflects a self-reinforcing immune–fibrotic feedback loop inflected by both local inflammatory pathways and systemic neuroendocrine factors. This integrated framework helps explain treatment resistance and highlights the need for therapeutic strategies targeting both immune signaling and fibroblast activity.

## 8. Future Directions

Future research should prioritize the identification of immune biomarkers that can predict keloid recurrence and therapeutic resistance. Persistent elevation of pro-inflammatory and pro-fibrotic cytokines such as IL-6, TNF-α, TGF-β, and IL-17 has been consistently observed in keloid tissue, underscoring the central role of immune dysregulation in disease chronicity and progression [9,28]. Most available data in the literature are cross-sectional, limiting these markers as associative rather than true predictors of disease recurrence. These biomarkers could help treatment guidance and selection, and follow-up strategies, moving keloid management beyond a standard procedure for all patients [10]. 

Altogether, systematic integration of stress contributors, depression, and anxiety assessments together with immune profiling and clinical outcomes can provide the answer to how neuroendocrine and immune pathways interact to influence scar persistence and treatment response [12].

## 9. Conclusions

Refractory keloids and hypertrophic scars should not be seen solely as technically challenging or treatment-resistant lesions. These should be looked at as manifestations of complex biological dysregulation. Increasing evidence shows that these scars arise from chronic immune activation, abnormal fibroblast upregulation and signaling, and sustained inflammatory signaling extending beyond the localized tissue abnormalities. It is important to remember that pathological scarring reflects a systemic-immune-neurocutaneous issue rather than just a single system dysfunction. This multifactorial interaction provides a plausible explanation for the high recurrence rates of refractory scars and keloids despite an appropriate therapeutic procedure. Advancing a multidisciplinary approach in clinical practice and the development of personalized strategies that address the underlying drivers of disease will be essential for improving long-term outcomes and reducing recurrence in refractory scarring.

## Figures and Tables

**Table 1:** Current therapeutic modalities for keloids and hypertrophic scars: mechanisms and key limitations.

Therapy	Mechanism of Action	Limitations
Surgical excision	Removes fibrotic tissue	High recurrence when used as a monotherapy
Corticosteroids	Suppress inflammation	Skin atrophy and hypopigmentation are common side effects
Radiation therapy	Inhibits fibroblast proliferation and collagen synthesis	Risk of long-term carcinogenesis
Laser therapy	Reduces vascularity and collagen remodeling	Requires multiple sessions
Silicone gel	Hydration	Requires prolonged therapy adherence
Cryotherapy	Induces cellular destruction	Hypopigmentation and pain side effects
Combination therapy	Targets multiple pathways simultaneously	Recurrence risk

## Data Availability

No new data were created or analyzed in this study.
